# Proprioceptive Neuromuscular Facilitation (PNF)-Integrated Gait Rehabilitation Following Total Hip Arthroplasty

**DOI:** 10.7759/cureus.57854

**Published:** 2024-04-08

**Authors:** Rishika Gabada, Swapna Jawade, Priya Tikhile

**Affiliations:** 1 Musculoskeletal Physiotherapy, Ravi Nair Physiotherapy College, Datta Meghe Institute of Higher Education and Research, Wardha, IND

**Keywords:** gait rehabilitation, aseptic osteonecrosis, rehabilitation program, physiotherapy intervention, total hip arthroplasty (tha), femoral head avascular necrosis (avn)

## Abstract

As a type of aseptic osteonecrosis, femoral head avascular necrosis (AVN) is characterized by abnormal blood flow that results in osteocyte death and femoral head degradation. Trauma, alcohol abuse, corticosteroid usage, and a few underlying medical disorders are common reasons. A 46-year-old farmer who had acute femoral head damage and left hip pain is described in this case study as having undergone total hip arthroplasty (THA). The systematic plan of the physiotherapy intervention included patient education, joint restoration, pain management, prevention of complications, strengthening, proprioception, endurance, and task-oriented motor relearning activities. Over the course of four weeks, the patient demonstrated improvements in functional outcomes and pain levels, highlighting the significance of a thorough physiotherapy approach in the management of AVN following THA. For the best possible patient results, this case study emphasizes the importance of early detection, diagnosis, and a well-coordinated rehabilitation program.

## Introduction

The femoral head's avascular necrosis (AVN) is a type of aseptic osteonecrosis characterized by a disruption in blood flow to the upper portion of the femoral head. Traumatic or non-traumatic events may give rise to this illness by inducing ischemia, which, in turn, causes AVN [1]. Alcohol misuse, hip joint fractures or dislocations, and corticosteroid treatment are frequent etiological variables contributing to this condition. Individuals who are physically active between the ages of 20 and 40 are the most vulnerable to this condition [2]. The lateral and medial circumflex branches of the profunda femoris artery, which arises from the femoral artery, are primarily responsible for supplying blood to the femoral head in adults and adolescents. Blockage to subchondral microcirculation, particularly in the retinacular vessels, can cause bone necrosis. This bone cell necrosis is linked to a higher risk of developing secondary osteoarthritis and hip range of motion restrictions because the damaged osteonecrotic region accumulates microfractures [3]. When fractures or dislocations in the femoral head restrict blood flow to that area, posttraumatic AVN results. Fractures that occur in the sub-capital area of the femoral neck are typically linked to AVN. Damage to this particular area disrupts the lateral epiphyseal artery connection, limiting blood supply to the femoral head [4]. The diagnosis of AVN is mostly based on a synergy between radiographic evaluations and clinical observations. Increasing pain, stiffness, and crepitus are common clinical indicators that usually appear after a period of minor symptoms [5]. A complete physical examination often reveals that patients have restricted hip range of motion, often with associated pain, particularly with forced internal rotation. When it comes to future diagnostic evaluations and treatment planning, these complaints are important markers for possible hip joint problems [6]. Improving results depends on early detection of bone necrosis indicators. Numerous imaging methods, including computed tomography (CT), magnetic resonance imaging (MRI), radionuclide exams, and X-rays, have shown promise in this domain. Radiography is a readily available and reasonably priced method for beginning the imaging examination of AVN [7]. The existence of a subchondral radiolucency, also called the crescent sign, in traditional radiography may be suggestive of a subchondral collapse [8]. In the later phases of AVN, CT and X-ray imaging show lesser sensitivity than MRI in identifying necrotic changes [9]. The goal of the conservative AVN treatment strategy is to improve hip function, prevent the femoral head from collapsing, reduce pain, and delay the development of necrotic alterations [9]. In the early stages of the condition (radiographic Steinberg stages 0-10), patients are usually treated without surgery. In general, this method is consistent with imaging results that fall into stages 0 and 1 of the Steinberg scale [10]. Joint-preserving treatments are generally used in the surgical management of AVN and the early stages of the condition, such treatments are usually recommended for younger patients. For those with extensive AVN, total hip arthroplasty (THA) becomes a suitable solution [11]. THA may be an appropriate solution for patients with severe discomfort, significant decline in hip function, and extensive femoral head collapse. To treat the aforementioned problems, this surgical operation involves replacing the hip's ball and socket with an artificial implant [12]. While younger patients may have limits following THA, such as activity constraints and possible revisions to the implant, most patients have positive outcomes, such as significant pain alleviation and hip function restoration [13]. The successful care of this illness has been the combination of physiotherapy alongside medicinal and surgical techniques. To reduce symptoms, improve functional independence, and improve the general quality of life for those who are dealing with this illness, physical therapy is essential [14]. After THA, individuals may encounter a protracted period - up to a year - during which they find it difficult to return to their previous state of functioning as long as they are not involved in an organized rehabilitation program. To prevent common problems like dislocation and thromboembolic illness, as well as to improve strength and walking speed, physiotherapy is essential for the management of these patients. In addition, physiotherapy after total hip arthroplasty encourages greater mobility and offers crucial instruction on exercises and safety measures for both hospital stay and post-discharge. Proprioceptive neuromuscular facilitation (PNF) techniques are often incorporated to enhance rehabilitation outcomes [15].

## Case presentation

Patient information

A 46-year-old man who works as a farmer started having pain in his right hip (over the greater trochanter [GT], laterally to GT and groin) eight months ago. The pain was insidious in onset and gradually progressive in nature. The pain was situated over the GT and posterolateral aspect, tracing along the sutures of the right hip joint, as well as affecting the anterior and lateral regions of the left hip joint. The onset of the pain was gradual, with a dull aching quality. The pain was continuous, with a severity rating of 3/10 on the visual analog scale (VAS) at rest and 8/10 during movement, particularly exacerbated when transitioning from supine to long sitting. Other aggravating factors included riding a bike and driving a car, prolonged standing, sitting with crossed legs, using the toilet, walking, crouching, and extended standing, all of which were found to be particularly painful. Resting provided relief from the pain. The pain was relieved upon resting. When the patient visited Acharya Vinoba Bhave Rural Hospital (AVBRH) to seek treatment, an X-ray was taken, and the results showed severe femoral head destruction. The patient subsequently underwent total hip arthroplasty as recommended by the surgeon. A thorough physiotherapy rehabilitation program was started after the surgery to help with the recovery process.

Clinical findings

**Figure 1:**
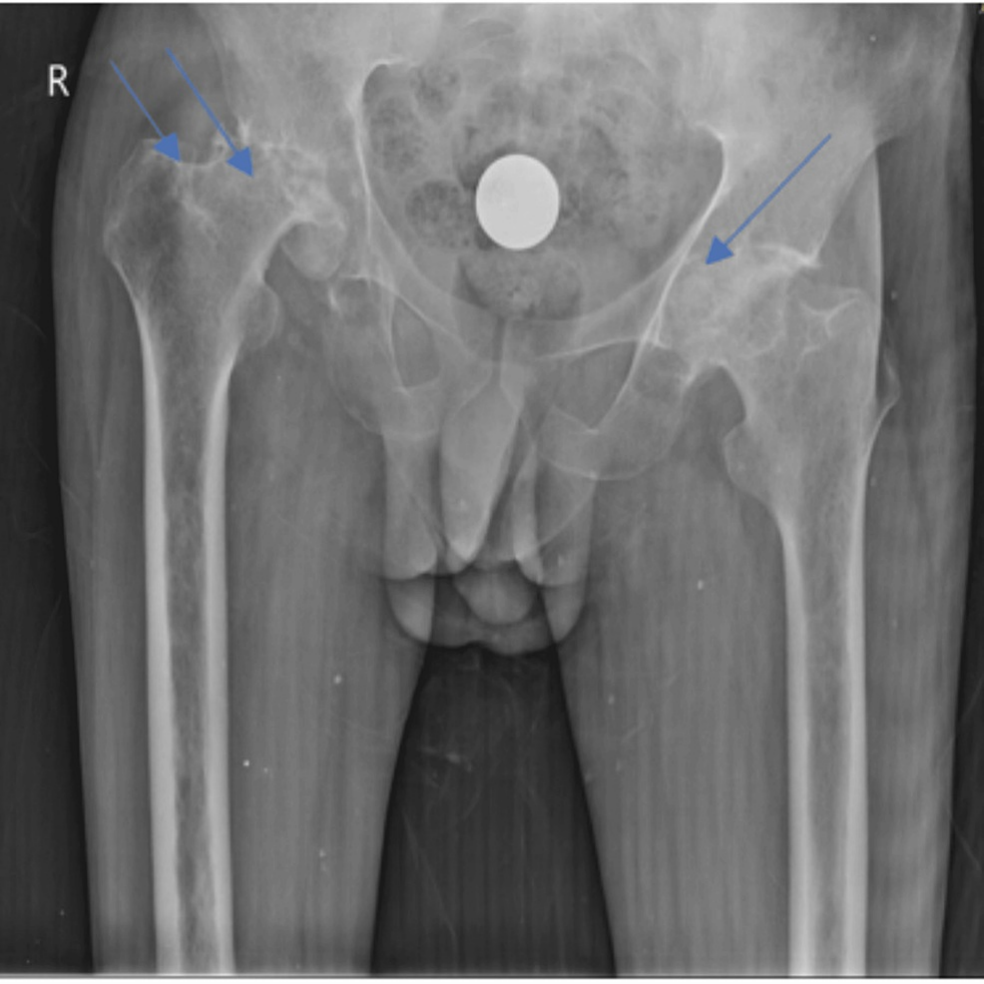
Preoperative X-ray of the pelvis and both hips showing (1) bilateral flattening and destruction with lytic changes of the femoral head; (2) loss of contour of the right and left femoral head; and (3) subchondral collapse and sclerosis of the bilateral femoral head.

**Figure 2:**
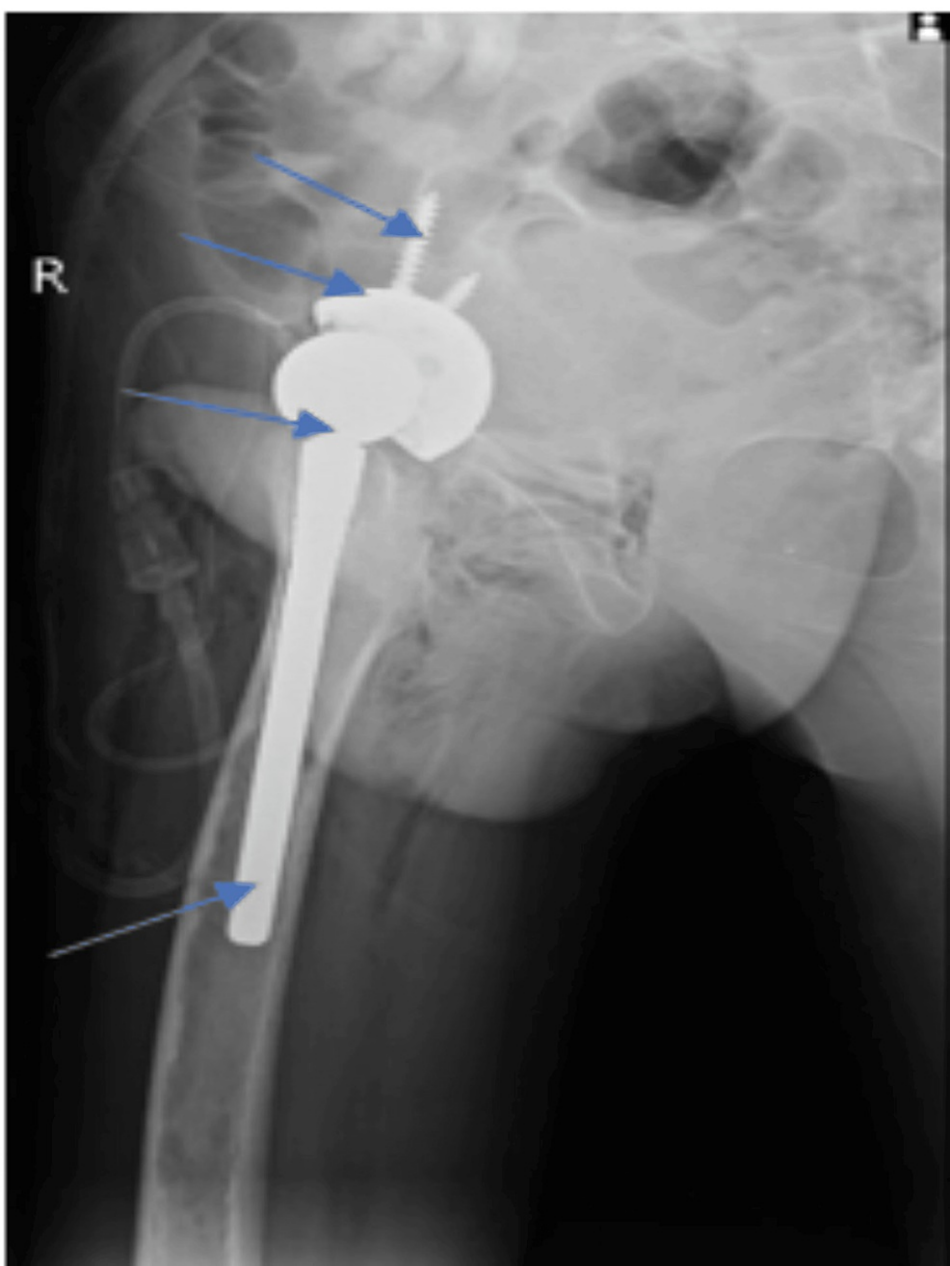
Postoperative X-rays of the uncemented total hip arthroplasty of the right hip joint showing a modular shell and hip component, bone screws, modular femoral head, and uncemented femoral stem.

The patient was conscious, cooperative, and fully oriented to time, place, and person. There were no evident indications of pallor, icterus, cyanosis, clubbing, lymphadenopathy, or peripheral edema. The patient had an ectomorphic body build, at 165 cm in height and 53 kg in weight; the computed body mass index (BMI) was 19.5, suggesting the patient's BMI was within the acceptable range. The temperature was afebrile. Before conducting the evaluation, the patient provided their express informed consent and was positioned in the supine lying position. On inspection, there was a dressing covering the right hip joint's posterolateral side, covering a healthy scar on the same surface. Mild swelling was evident around the suture site and approximately 1.5 cm laterally below it. The inspection findings were confirmed through palpation. Examination of the open wound and scar on the postero-lateral surface of the right hip joint revealed a healthy scar approximately 8 cm in length, curving from the posterior edge of the GT. Local temperature in the area was observed to be elevated compared to the opposite left side. Tenderness was observed, with Grade 2 tenderness present over the suture site and lateral aspect of the right hip joint, and Grade 1 tenderness over the GT, as well as the anterior and lateral aspects of the left hip joint. Following a close examination, it was discovered that the patient had an antalgic gait and Trendelenburg gait. Figures 1-2 show the pre- and postoperative X-rays of the patient. Additionally, the ROM of the hip, measured with the assistance of a goniometer, was markedly restricted, and any movement of the hip caused agony. The ROM and manual muscle testing (MMT) results are shown in Tables 1-2, respectively. Furthermore, there was a noticeable discrepancy in limb length, with the left appearing shorter than the right side, which was measured with the help of inch tape, as shown in Table 3. The girth measurement is shown in Table 4. Upon observation, distinct deviations in the individual's gait were noted. There was a discernible deviation in the trunk's trajectory, specifically leaning toward the left leg during the stance phase. This asymmetrical weight distribution contributes to an observable imbalance in the overall gait pattern. Furthermore, the stride length and step length were noticeably reduced, while the width increased, resulting in not only a restricted gait speed but also impinging on the individual's ability to comfortably cover an adequate walking distance. This gait deviation, characterized by altered trunk alignment and reduced stride length, highlights the impact of these biomechanical alterations on the person's mobility and functional ambulation. The complete incident's timeframe is depicted in Table 5.

**Table 1:** Range of motion.

Movement	On admission (°)	At discharge (°)
Hip flexion	NA	0-85
Hip extension	0-7	0-11
Hip abduction	0-20	0-30
Hip adduction	0-10	0-17
Knee flexion	0-95	0-110
Knee extension	95-0	110-0
Ankle plantarflexion	0-40	0-45
Ankle dorsiflexion	0-15	0-20

**Table 2:** Manual muscle testing.

Muscles	On admission	At discharge
Hip flexors	NA	3+/5
Hip extensors	2+/5	3/5
Hip abductors	2-/5	2/5
Hip adductors	NA	3+/5
Knee flexors	3+/5	4/5
Knee extensors	3/5	4/5
Ankle plantarflexors	4+/5	5/5
Ankle dorsiflexors	4+/5	5/5

**Table 3:** Limb length measurement. GT, greater trochanter; LC, lesser trochanter

Limb length measurement	Affected side (Right)	Nonaffected side (Left)	Difference
True length	86 cm	84.5 cm	1.5 cm
Apparent length	92.5 cm	91 cm	1.5 cm
Segmental length			
GT to LC of the femur	42 cm	40 cm	2 cm
LC of the tibia to lateral	35 cm	36 cm	1 cm

**Table 4:** Girth measurement.

Girth measurement	Right	Left	Difference
From the base of the patella (quadriceps)			
2 inch	30.5 cm	31 cm	0.5 cm
4 inch	32.5 cm	33 cm	0.5 cm
6 inch	37 cm	35 cm	2 cm
From the apex of the patella (calf muscle)			
2 inch	28 cm	28 cm	0 cm
4 inch	26 cm	26 cm	0 cm
6 inch	25 cm	25 cm	0 cm

**Table 5:** Timeline. AVBRH, Acharya Vinoba Bhave Rural Hospital

October 21, 2023	The patient visited AVBRH
October 22, 2023	Investigations conducted
October 25, 2023	Surgery carried out
October 26, 2023	Physiotherapy started

Therapeutic intervention

**Figure 3:**
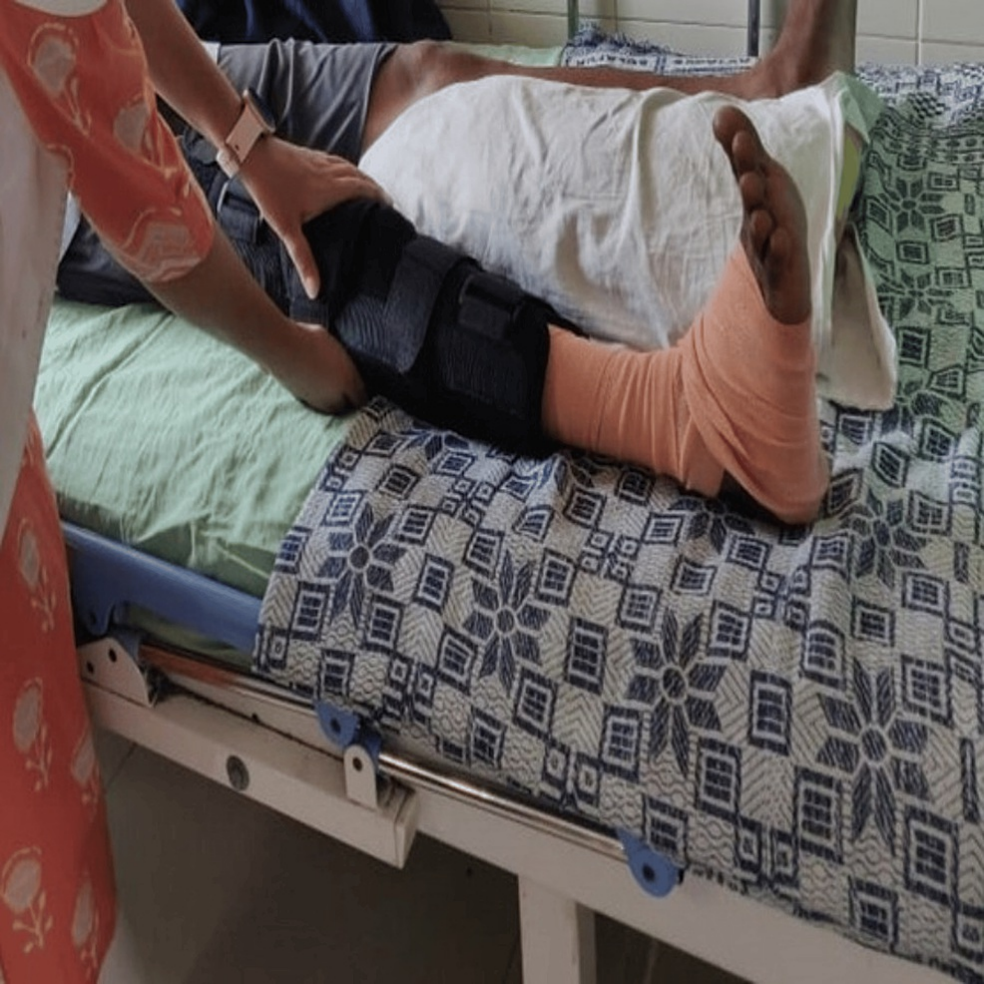
The patient performing static quads (long knee brace used for stability).

**Figure 4:**
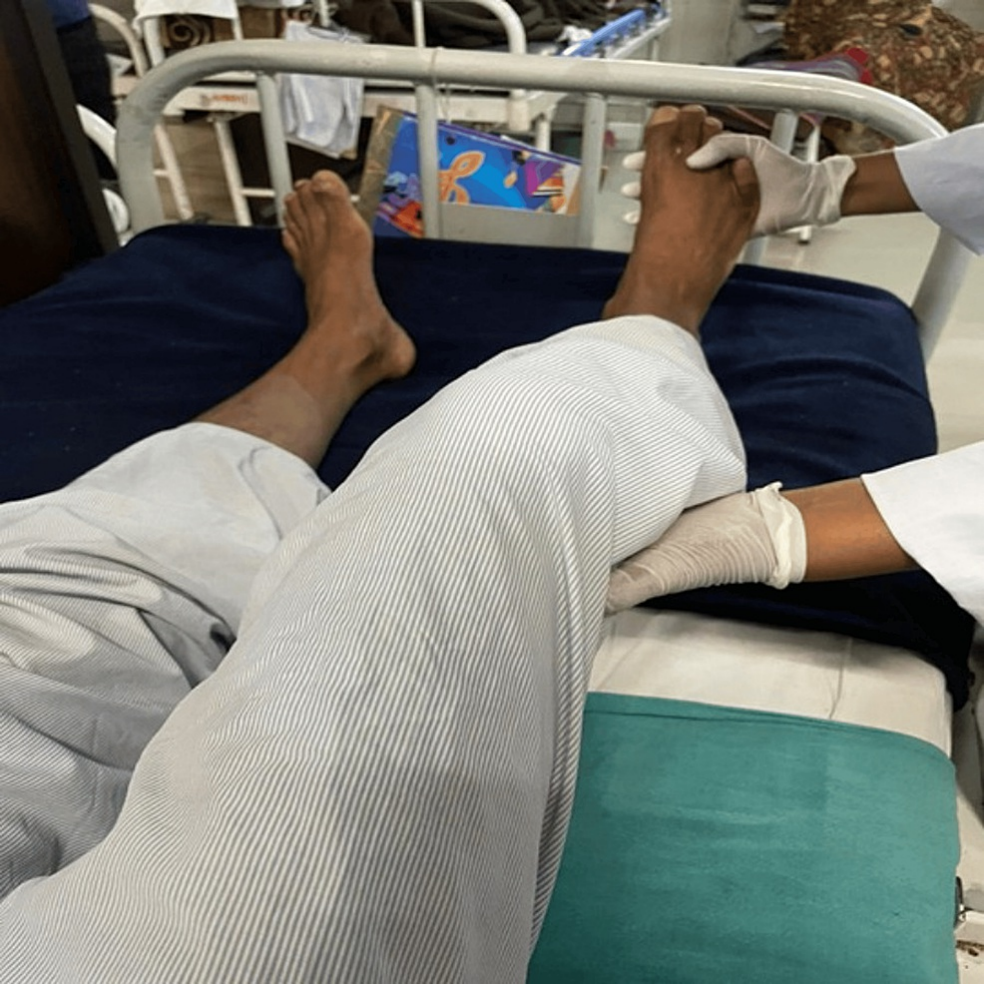
Functional leg pattern D1, extension/abduction/internal rotation with knee extension.

The goal-oriented physiotherapy protocol is given in Table 6. Physiotherapy sessions included strengthening exercises and proprioceptive neuromuscular facilitation (Figures 3-4, respectively).

**Table 6:** Goal-oriented physiotherapy protocol. PNF, proprioceptive neuromuscular facilitation

Goals	Intervention	Treatment regimen
Phase 1 (day 1-2 weeks)		
Patient education	Giving the patient and their family complete information about their condition and other necessary information, appropriate safety measures, ways to prevent dislocation, and the role that physical therapy plays in the healing process.	Counseling sessions focused on addressing the benefits and importance of walking, being physically active, and keeping appropriate body alignment.
To reduce inflammation and relieve pain	Cryotherapy	Each session 10-15 minutes, twice a day
To help reduce the likelihood of developing further respiratory and circulatory system issues	Breathing exercises, ankle toe movements, spirometer, active range-of-motion (ROM) exercises on the unaffected side, heel slides, and hip abduction in a gravity-eliminated position	Each set of 10 repetitions
Positioning	To prevent hip adduction, a pillow should be positioned between both legs.	Ought to be carried out either horizontally while sitting or reclined on the bed
Functional mobility	Improving bed mobility, enhancing transfer techniques, executing gait training with appropriate assistive devices on level ground, and improving stair navigation abilities	Two rounds of a 15 m hallway
Phase 2 (two to six weeks)		
Begin to restore the ROM	Continuing with all Phase 1 exercises, including joint mobilizations and stretching exercises for the hamstring, gastrosoleus, and quadriceps muscles.	Each set of 10 repetitions
Strengthening	Phase progression: Begin with seated hip flexion and quad/glut/ham sets. Adjusting hips to a standing flexion, abduction, and adduction. Following antigravity hip exercises and SLRs. The last phase culminates with closed-chain workouts such as mini-lunches, step-ups, and mini-squats.	10 repetitions x 1 set, held for 5 seconds for 3 weeks, then 10 seconds for 4 weeks, and finally 15 seconds for 5 weeks. Five repetitions in a set
Proprioception	Performing weight-shifting exercises in a single-leg stance	10 reps x 1 set and 5 reps x 1 set
Endurance	Stationary cycling with minimal resistance three to four weeks after surgery	
Functional mobility	To emphasize a natural gait pattern, use appropriate gait training devices and add appropriate devices for stair training as well.	
Gait-related PNF pattern training	Execution of the following PNF patterns to strengthen hip adductors and abductors: (1) leg pattern: knee extension, abduction, and internal rotation; (2) anterior elevation of the contralateral pelvis; (3) diagonal bridging in the manner of weight-bearing exercises by hip extension/abduction.	Three times a week
Phase 3 (6-12 weeks)		
Restore the average lower limb strength, especially the regular quadriceps function, and return to basic daily tasks at a minimal level of functioning.	ROM - Continuing phase 1 and 2 exercises Strengthening - Continue with phase 2 exercises- as tolerated, adding and increasing the resistance	twice a day for seven weeks, 10 repetitions for one set of ten minutes each.
Proprioception	Single leg stance: static balance on Bosu/wobble board/foam/etc. Adding gentle agility exercises (i.e., tandem walking, sidestepping, backward walking)	Twice a day for seven weeks, 10 repetitions for one set of 10 minutes each.
Endurance	Maintain a cycling regimen while introducing mild-to-moderate resistance as tolerated.	Ten repetitions for one set of 10 minutes
Exercises for rehabilitation that target PNF patterns, with a focus on improving motor skills; exercises emphasize posture, stepping, step length, and cadence.	From a supported stance in parallel bars, the exercises proceeded to (1) right arm pattern: extension/abduction/internal rotation; (2) right-side pelvic anterior elevation facilitation; and (3) left-side hip extension and abduction. During stair-stepping, on the left side, resisted hip extension, abduction, and internal rotation were applied during the gait phase at the terminal stance, while right pelvic anterior elevation occurred. For outside walking as part of a home workout routine, walking against resistance while holding a stick was advised.	Three times a week

Follow-up and outcome measures 

Table 7 shows the follow-up and outcome measures that were taken pre- and postintervention.

**Table 7:** Pre- and post-physiotherapy intervention outcome measures.

Scales	Preintervention	Postintervention
Numerical pain rating scale	9/10	4/10
Harris hip score	50/100	80/100
Lower extremity functional scale	19/80	66/80

## Discussion

This debilitating condition can be caused due to many things, including trauma, steroid use, and alcoholism, or it may develop idiopathically. AVN of the femoral head (AVNFH) is defined by a reduced blood supply to the femoral head, which causes bone deterioration and eventual collapse [16]. In this case, the patient exhibited pain in the left hip region and had trouble getting upstairs, walking, crouching, and carrying out everyday activities. AVN of the femur was identified, and a total hip replacement (THR) was chosen as the treatment for the cause of the issue. A well-thought-out and well-coordinated strategy for physiotherapy intervention has been created, starting with basic and gentle exercises and working up to strength-building activities, ultimately concluding with gait training initially with non-weight-bearing. According to the study carried out by Pierce et al., analysis indicates that following THR, patients with hip osteoarthritis benefited significantly in the short term from physiotherapy therapies in terms of mobility, muscle strength, health-related quality of life, and physical function [17]. According to Keizer et al., individuals frequently have persistent deficits in proprioceptive awareness and balance following complete joint replacement surgery. Functionality can be severely limited by these problems, which can result in changed gait patterns, trouble walking, and trouble keeping good posture. Because of this, rehabilitation programs stress how crucial it is to include proprioceptive and balance training to treat these long-term impairments and improve overall recovery results [18]. This case study aims to elucidate a comprehensive care plan for patients undergoing THA due to AVN and underscore the importance of physical therapy-assisted rehabilitation in their recovery. THA for AVN differs from standard THA in several aspects, primarily in the underlying pathology and the specific considerations required for rehabilitation. AVN is often associated with compromised bone quality and altered hip biomechanics, necessitating a tailored approach to therapy and rehabilitation. According to the study carried out by Gilbey et al., PNF was used in conjunction with gait rehabilitation training to restore altered movement patterns throughout the patient's everyday activities. This technique not only addressed structural deficiencies but also produced effects related to motor learning. PNF patterns were deliberately used to mimic and reproduce functional tasks that are frequently seen in sports and daily life. In this specific example, the customized PNF-based therapy that included important motor learning components turned out to be a useful and successful strategy [19].

## Conclusions

This case study demonstrates the effectiveness of comprehensive physiotherapy intervention, including PNF techniques, in the rehabilitation of a 46-year-old farmer following THA due to AVN. The patient showed significant improvements in functional outcomes and pain levels over four weeks, highlighting the importance of a well-coordinated rehabilitation program. PNF-integrated gait rehabilitation played a crucial role in restoring normal movement patterns and improving overall outcomes. This case emphasizes the value of early detection, diagnosis, and a holistic physiotherapy approach in managing AVN following THA.
